# Boron Intake and 24-hour Urinary Excretion in Long-term Omnivores, Vegetarians and Vegans: a Cross-sectional Study

**DOI:** 10.1007/s12011-026-05297-x

**Published:** 2026-08-12

**Authors:** Frieda Stübing, Stefanie Kowarschik, Christian Koeder, Johanna Dürrschnabel, Roman Huber, Maximilian Andreas Storz

**Affiliations:** 1 Department of Internal Medicine II, Centre for Complementary Medicine, Medical Center - University of Freiburg, Faculty of Medicine, University of Freiburg, Freiburg, Germany; 2 Independent researcher, Ellwangen, 73479 Germany

**Keywords:** Nutrition, Vegetarian, Vegan, Boron, Trace elements, Diet

## Abstract

The trace element boron is a bioactive and potentially essential element for humans with a broad range of physiological effects. While dietary sources of boron are well known, significant knowledge gaps remain regarding boron intake in special dietary patterns. We estimated dietary boron intake and quantified urinary boron excretion in long-term lacto-ovo-vegetarians, vegans and omnivores in Germany. Dietary intake was estimated using weighed food diaries linked to existing databases. Boron excretion was measured using 24-hour urine samples. Differences between diet groups were compared using non-parametric tests (Kruskal–Wallis *H* test). The sample included 89 participants, including 28 lacto-ovo-vegetarians and 29 vegans. Vegans had the highest median boron intake (1913.38 (interquartile range: 1467.11-2615.14) µg/d), followed by lacto-ovo-vegetarians (1654.36 (1249.75-2366.01) µg/d) and omnivores (1515.35 (944.33-2067.52) µg/d). Interindividual boron intake varied largely, and ranged from 657.09 to 5835.74 µg/d. No participant exceeded the European Food Safety Authority tolerable upper intake level for boron (10 mg/day). Creatinine-corrected urinary boron excretion was highest in vegans (1559.20 (1150.62-2057.23) µg/g creatinine), followed by lacto-ovo-vegetarians (1235.40 (877.96-1814.61) µg/g creatinine) and omnivores (919.34 (639.18-1380.97) µg/g creatinine). Strong correlations between boron intakes and 24-hour urinary excretion were found in vegans (*r* = 0.79) and omnivores (*r* = 0.74) with *p*-values < 0.001 for both. The correlation in lacto-ovo-vegetarians was less pronounced (*r* = 0.43). A higher urinary boron excretion was associated with a higher excretion of potassium in all dietary patterns and specifically phosphorus in vegans (*r* = 0.57, *p* < 0.001). Our data suggest that boron intake varies across different dietary patterns. We provide first insights in boron status in German lacto-ovo-vegetarians and vegans.

**Supplementary Information:**

The online version contains supplementary material available at https://doi.org/10.1007/s12011-026-05297-x.

## Background

Numerous studies over the past three decades have suggested that the trace element boron is a bioactive, beneficial and potentially even essential element for humans [1–4]. Boron has a broad range of physiological effects [3], and was associated with an improved bone health, central nervous system function and arthritis alleviation as well oxidative stress modulation in both preclinical and clinical studies [1, 5–8].

There are currently no established Recommended Dietary Allowances (RDAs) or Adequate Intakes (AIs) for boron because a definite human requirement is yet to be established [9–11]. While very high boron concentrations are toxic to the human body [3, 9], a dietary intake ranging from 1 to 13 mg per day might be acceptable [10]. In typical Western populations, dietary boron intakes usually range from 0.5 to 3 mg per day [9, 12]. Diets rich in plant foods (e.g., vegetables, fruits, nuts and seeds) have been associated with higher boron intakes [9, 13]. Boron from such dietary sources is efficiently absorbed and predominantly excreted by the kidneys. Other important dietary boron sources than plant foods include beverages (mainly coffee and wine) and milk products [4].

Pioneering expert reviews by FH Nielsen suggested that a low boron intake “is a relevant nutritional concern”, which may be prevented through dietary modifications [14]. Notably, Bartusik-Aebisher et al. recently emphasized that despite numerous epidemiological and toxicological studies, significant knowledge gaps remain regarding boron and its long-term effects on human health [9].

To the best of our knowledge, boron intake and excretion in diets that maximize boron-rich food sources (such as plant foods as part of a vegan diet and bovine milk in a lacto-ovo-vegetarian diet) has rarely been examined [15]. In fact, we are unaware of any study that specifically investigated boron intake and excretion in long-term vegans in Germany, who exclusively rely on plant-based foods and avoid animal-sourced foods to meet their nutrition goals. Against this background, the present study sought to quantify boron intakes and excretion using weighed food records and 24-hour urinary samples in long-term lacto-ovo-vegetarians and vegans from the German LAKE (Load of Acid to Kidney Evaluation) study [16]. Boron intake and excretion were contrasted to that of omnivores in a sample of young and healthy individuals. This study was conducted under the hypothesis that vegans would have a much higher boron intake than lacto-ovo-vegetarians and omnivores, as reflected in their dietary records and by their urinary excretion. As a secondary (exploratory) goal, we sought to identify potential associations between boron excretion and the urinary excretion of other chemical elements in all dietary patterns.

## Methods

### Data Source

Data and urinary samples were drawn from the German LAKE study [16]. The German LAKE study is a cross-sectional, Freiburg-based study which sought to validate a translated version of the German LAKE score in a three-armed study in a healthy population comprised of individuals following one of three different dietary patterns since at least 12 months [16]. These three dietary patterns included an omnivorous diet, a lacto-ovo-vegetarian diet (which excludes meat, poultry and fish but includes eggs and/or dairy products [17]) and a vegan diet (which excludes all animal-sourced foods [17]). Details regarding the LAKE study may be found in the original publication [16]. In brief, participants in the German LAKE study were asked to complete weighed food diaries on two consecutive days (day 1 and day 2) and a single 24-hour urine collection. Twenty-four-hour urine was collected on day 2 using an established protocol (see below) [16]. The LAKE study had the purpose to validate the German LAKE score – a clinical questionnaire to assess the potential renal acid load from diet.

The ethical committee at the University Medical Center of Freiburg approved the LAKE study in August 2024 (approval number: 24–1337-S1). The study was registered at the German Clinical Trial Register (DRKS00035243) on 8 October 2024 and began on 30 October 2024 [18]. Urinary samples were collected between October 2024 and January 2025. Samples were frozen at −80 °C and stored appropriately [16]. A batch of those samples was used for this secondary analysis in April 2026.

### Outcomes of this Secondary Analysis

The main aim of this secondary (follow-up) analysis was to examine dietary boron intake in omnivores, lacto-ovo-vegetarians and vegans from the German LAKE study. We aimed to investigate whether dietary boron intake differed between the three dietary patterns examined in this study. Further to that, we aimed to examine associations between dietary boron intake and urinary boron excretion using 24-hour-urine samples. Finally, we sought to explore associations of boron excretion with other chemical elements determined in the 24-hour-urine samples.

### 24-hour Urine Samples and Boron Analyses

Participants provided a single 24-hour urinary collection on the second day of the study. Urine collection began in the morning (after participants emptied their bladder for the first time) using brown-colored 3 L Sarstedt urine containers [16]. During the collection period, these containers had to be stored in a refrigerator or outside (the study was conducted in winter with cold outside temperatures). The collection ended in the morning of the following day (and included the first morning micturition); samples were processed immediately thereafter. All study-related laboratory analyses except boron were performed at the accredited Central Laboratory of the University Medical Center of Freiburg (Institute of Clinical Chemistry and Laboratory Medicine, Medical Centre – University of Freiburg, Hugstetter Str. 55, 79106 Freiburg im Breisgau, Germany). Chemical elements were measured using Cobas^®^ 8000 modular analyzer series (Roche Diagnostics). Urinary boron excretion was determined at the LGM Laboratory in Hamburg, Germany (LGM HH, Grandweg 64, 22529 Hamburg, Germany). Urinary boron concentrations were measured using Inductively Coupled Plasma Mass Spectrometry (ICP-MS; PerkinElmer NexION 2000). Prior to analysis, urine samples were diluted ten-fold using a laboratory-prepared dilution solution. Boron quantification was performed using a four-point calibration curve with linear regression through zero. Calibration standards and dilution solutions were prepared specifically for boron analysis. Internal standardization with yttrium and terbium was applied to correct for variations in instrument response and potential matrix effects. The analytical performance of the method was monitored using Seronorm™ Trace Elements Urine quality control material (Invicon) at two concentration levels (REF 210605 and REF 210705). The quality control samples were analyzed together with the study samples to ensure analytical accuracy and precision. The analytical detection limit for boron was 0.21 µg/L.

### Weighed Food Diaries

Dietary intake in this study was captured using two prospective 24-hour weighed food diaries [16]. For this, we asked all participants to list all consumed food items and to quantify them to the nearest 0.1 g or ml, respectively [16]. Participants received calibrated precision scales and a detailed in-person instruction for this method. A detailed description of this process may be found in the original study as well as in other studies in vegans conducted by our research group [19, 20]. This approach was selected because weighed food diaries are considered the gold standard in nutrition research and because they capture foods that are popular among lacto-ovo-vegetarians and vegans (e.g., tempeh, soy, etc.) but that are not routinely included in standard food frequency questionnaires. While standard food frequency questionnaires may not capture all food items relevant for vegan diets, they are easier to administer, more cost-effective and they do not require weighing of foods.

We then used NutriGuide^®^ plus software (Version 4.9, Nutri-Science GmbH, 77756 Hausach, Germany) for dietetic analyses. Notably, standard dietetic software does not automatically estimate boron intake. Instead, we compiled the boron content of various foods from previous databases/publications and constructed a master file which contains the boron content of *n* = 212 foods [4, 21, 22]. We thereby relied heavily on US-based data from Rainey et al. [4] and supplemented the database with boron content data for typical foods in vegans (e.g., soy, algae) which was provided by Choi and Jun [21]. Average boron intake of both 24-hour recalls was then estimated. Boron content data for tap water from the Freiburg area was used for this study. For local mineral waters, we used the mean boron content of 381 German mineral waters determined by Seidel et al. in 2021 [23].

### Inclusion and Non-Inclusion Criteria

Inclusion and non-inclusion criteria were initially designed for the validation of the LAKE score and unrelated to this secondary analysis [16, 18]. Thus, these criteria were initially designed to minimize the risk of malabsorption-related confounders and other aspects interfering with mineral uptake (which is of high relevance for the LAKE scare). Healthy individuals between 18 and 60 years of age who agreed to complete weighed food diaries on two days were recruited. Great attention was paid to dietary adherence and only individuals who self-reported not having changed their diet over the last 12 months were considered. During the course of the study, participants were explicitly asked to avoid any dietary supplements.

Non-inclusion criteria included a recent intake of proton pump inhibitors, diuretics or uricosurics as well as a history of diseases of the gastrointestinal tract. Pregnant or breastfeeding women were not included. Likewise, we did not include individuals with a BMI outside the range of 18.5 kg/m² to 30 kg/m². Notably, the main study also included some food-related exclusion criteria which could be of relevance for the current study. Participants who reported a coffee consumption ≥ 1 L/day, a consumption of ≥ 100 g of fresh berries/day or an intake of algae (≥ 20 g/day) were not included. These criteria were initially introduced to avoid the inclusion of individuals with extremely low urinary potential renal acid load values from the study.

### Statistical Analysis

The statistical analysis was performed by MAS in Stata19 (StataCorp. 2025. Stata Statistical Software: Release 19. College Station, TX: StataCorp LLC.) in June 2026. The main study was powered to assess correlations between the LAKE score and the potential renal acid load score [16]. With an estimated Spearman’s rank correlation coefficient of 0.5 and a power of 0.80, 23 participants per dietary group would have been required to detect a significant correlation (alpha was set at 0.05). We anticipated a drop-out rate of at least 15% and thus recruited at least 27 participants per group. Notably, the power calculations were initially not performed for the outcome of this secondary analysis (boron intake and excretion).

Boron intake and excretion were compared across the three dietary groups. When comparing boron excretion, we used absolute values (in µg/24-h), urinary concentration values (in µg/l) and creatinine-corrected values (in µg/g creatinine). The latter was done to account for known differences in urinary creatinine values between vegans and omnivores [16]. Descriptive statistics included means (± standard deviations) for normally distributed data and medians (interquartile ranges) for non-normally distributed data. Histograms, box plots and Stata’s Shapiro–Wilk test were used to test for normality [16]. Pearson’s correlation coefficients (*r*) and Spearman’s correlation coefficients (*ρ*) were used to examine potential associations between boron intake and excretion. Results were visualized with scatter plots and strip plots [24]. For the between group comparisons, we used parametric and non-parametric tests as applicable. The Kruskal–Wallis *H* test was used to determine if there were statistically significant differences between the three groups in non-normally distributed independent variables of interest.

## Results

The final sample comprised *n* = 89 individuals who successfully completed the German LAKE study and who provided both 24-hour urinary samples and dietary recall data. A participant inclusion flowchart is shown in Supplementary Fig. 1. Table 1 displays sociodemographic sample characteristics, which did not differ between groups. The sample was mainly comprised of students, with a median age of 23 years. Urinary creatinine values as well as excretion rates of all chemical elements investigated in this study are displayed in Table 2.

**Table 1:** title: Sample characteristics of *n* = 89 individuals participating in the German LAKE study

Variable	All participants *n* = 89	OM *n* = 32	LOV *n* = 28	VEG *n* = 29	*p*-value
**Sex**					*p* = 0.240
Males	*n* = 25 (28.09%)	*n* = 12 (37.50%)	*n* = 5 (17.86%)	*n* = 8 (27.59%)	
Females	*n* = 64 (71.91%)	*n* = 20 (62.50%)	*n* = 23 (82.14%)	*n* = 21 (72.41%)	
**Age** (years)	23 (22–26)	23 (22–25.5)	23 (22–24)	25 (22–28)	*p* = 0.434
**BMI** (kg/m²)	21.85 (21–23.31)	22.18 ± 1.63	22.19 ± 2.20	22.40 ± 2.14	*p* = 0.890
**Race/Ethnicity**					*p* = 0.830
Caucasian	*n* = 86 (96.63%)	*n* = 30 (93.75%)	*n* = 27 (96.43%)	*n* = 29 (100%)	
Turk	*n* = 1 (1.12%)	*n* = 0 (0%)	*n* = 1 (3.57%)	*n* = 0 (0%)	
Asian	*n* = 1 (1.12%)	*n* = 1 (3.13%)	*n* = 0 (0%)	*n* = 0 (0%)	
Portuguese	*n* = 1 (1.12%)	*n* = 1 (3.13%)	*n* = 0 (0%)	*n* = 0 (0%)	
**Educational level**					*p* = 0.184
Lower secondary education	*n* = 1 (1.12%)	*n* = 0 (%)	*n* = 0 (0%)	*n* = 1 (3.45%)	
German Abitur	*n* = 76 (85.39%)	*n* = 30 (93.75%)	*n* = 25 (89.29%)	*n* = 21 (72.41%)	
University degree	*n* = 11 (12.36%)	*n* = 2 (6.25%)	*n* = 3 (10.71%)	*n* = 6 (20.69%)	
Vocational school degree	*n* = 1 (1.12%)	*n* = 0 (%)	*n* = 0 (0%)	*n* = 1 (3.45%)	
**Marital status**					*p* = 1.000
Single	*n* = 87 (97.75%)	*n* = 31 (96.88%)	*n* = 28 (100%)	*n* = 28 (96.55%)	
Married	*n* = 2 (2.25%)	*n* = 1 (3.13%)	*n* = 0 (0%)	*n* = 1 (3.45%)	
**Occupation**					*p* = 0.616
Student	*n* = 74 (83.15%)	*n* = 28 (87.5%)	*n* = 24 (85.71%)	*n* = 22 (75.86%)	
Industry	*n* = 2 (2.25%)	*n* = 0 (0%)	*n* = 0 (%)	*n* = 2 (6.90%)	
Health professional	*n* = 4 (4.49%)	*n* = 1 (3.13%)	*n* = 1 (3.57%)	*n* = 2 (6.90%)	
Apprentice	*n* = 6 (6.74%)	*n* = 3 (9.38%)	*n* = 2 (7.14%)	*n* = 1 (3.45%)	
Voluntary social year	*n* = 1 (1.12%)	*n* = 0 (%)	*n* = 1 (3.57%)	*n* = 0 (%)	
Human resource officer	*n* = 1 (1.12%)	*n* = 0 (%)	*n* = 0 (%)	*n* = 1 (3.45%)	
Gardener	*n* = 1 (1.12%)	*n* = 0 (%)	*n* = 0 (%)	*n* = 1 (3.45%)	
**Physical activity level**					*p* = 0.944
Inactive	*n* = 8 (8.99%)	*n* = 2 (6.25%)	*n* = 3 (10.71%)	*n* = 3 (10.34%)	
Minimally active	*n* = 41 (46.07%)	*n* = 16 (50%)	*n* = 13 (46.43%)	*n* = 12 (41.38%)	
HEPA active	*n* = 40 (44.94%)	*n* = 14 (43.75%)	*n* = 12 (42.86%)	*n* = 14 (48.28%)	

modified from [16]. HEPA = Health-enhancing physical activity. OM = Omnivores. LOV = Lacto-ovo-vegetarians. VEG = Vegans

**Table 2:** title: Urinary excretion of boron and other chemical elements in *n* = 89 individuals participating in the German LAKE study

Variable	All participants *n* = 89	OM *n* = 32	LOV *n* = 28	VEG *n* = 29	*p*-value
Urinary volume (ml/24-h)	2533.01 ± 649.25	2424.38 ± 519.91	2519.93 ± 658.37	2665.52 ± 758.77	*p* = 0.351
Urinary creatinine (g/24-h)	1.13 (0.98–1.34)	1.25 (1.10–1.58)	1.07 (0.86–1.32)	1.10 (0.95–1.18)	*p* = 0.005
Boron excretion (µg/24-h)	1415.39 (1053.61–2169.99	1159.06 (875.72–1908.27)	1498.50 (1025.20–2000.31)	1848.59 (1326.51–2397.14)	*p* = 0.019
Boron excretion (µg/g Cr.)	1214.56 (863.96–1808.69)	919.34 (639.18–1380.97)	1235.40 (877.956–1814.61)	1559.20 (1150.62–2057.23)	*p* < 0.001
Calcium (g/24-h)	0.12 (0.07–0.16)	0.14 ± 0.06	0.12 (0.08–0.17)	0.09 ± 0.04	*p* < 0.001
Calcium (g/g Cr.)	0.10 (0.06–0.13)	0.11 ± 0.04	0.12 ± 0.07	0.08 ± 0.03	*p* = 0.007
Magnesium (g/24-h)	0.10 ± 0.03	0.10 ± 0.04	0.10 ± 0.04	0.10 ± 0.03	*p* = 0.973
Magnesium (g/g Cr.)	0.09 ± 0.03	0.08 ± 0.03	0.09 ± 0.03	0.09 ± 0.03	*p* = 0.017
Potassium (g/24-h)	2.69 (2.10–3.14)	2.58 ± 0.81	2.53 ± 0.95	2.95 ± 1.12	*p* = 0.264
Potassium (g/g Cr.)	2.34 (1.64–2.94)	1.97 ± 0.71	2.45 ± 0.88	2.63 (2.00–3.26)	*p* = 0.009
Phosphorus (g/24-h)	0.66 (0.51–0.87)	0.86 (0.74–0.98)	0.62 ± 0.25	0.58 ± 0.23	*p* < 0.001
Phosphorus (g/g Cr.)	0.60 ± 0.19	0.67 ± 0.19	0.59 ± 0.18	0.52 ± 0.18	*p* = 0.006

*OM* Omnivores, *LOV *Lacto-ovo-vegetarians, *VEG *Vegans

**Fig. 1:**
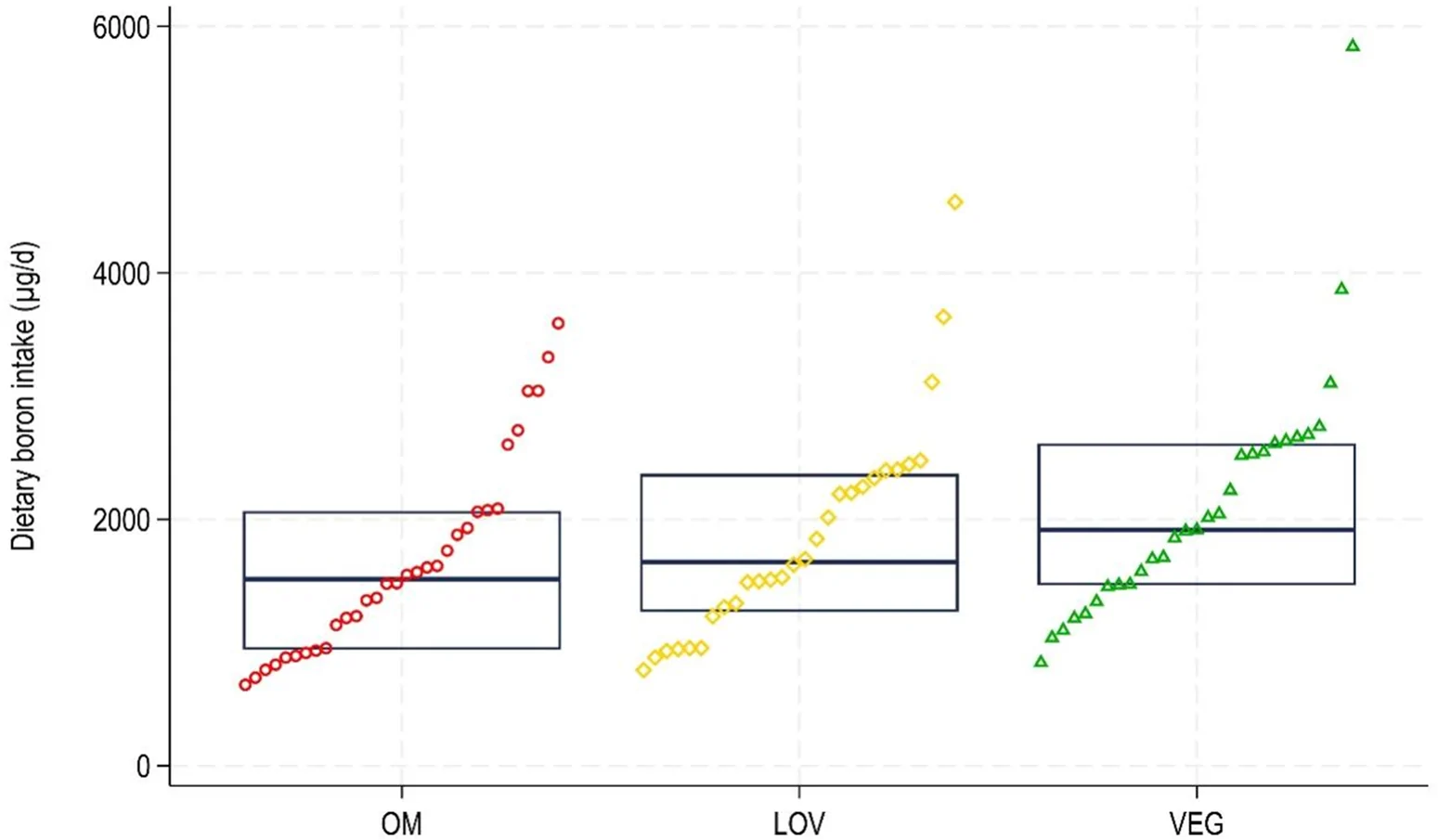
Dietary boron intake in *n* = 89 individuals participating in the German LAKE study by dietary group. Abbreviations: OM = Omnivores. LOV = Lacto-ovo-vegetarians. VEG = Vegans. Median boron intakes were as follows: omnivores: 1515.35 (944.33–2067.52) µg/d; lacto-ovo-vegetarians: 1654.36 (1249.75–2366.01) µg/d; vegans: 1913.38 (1467.11- 2615.14) µg/d. The between group difference was not statistically significant at *p* = 0.104

Figure 1 displays dietary boron intakes in the study sample and by dietary group in a strip plot. Vegans had the highest median dietary boron intake, followed by lacto-ovo-vegetarians and omnivores. A high variance was found in the whole sample and even within the distinctive dietary patterns. Boron intakes ranged from 657.09 µg/d to 5835.74 µg/d per day. The between group difference was not statistically significant (*p* = 0.104). No participant exceeded the EFSA (European Food Safety Authority) tolerable upper intake level for boron, which is currently set at 10,000 µg/d [25]. As discussed earlier, energy intake did not differ between the three dietary groups [16]. As such, no energy-adjusted boron intakes were calculated.

**Fig. 2:**
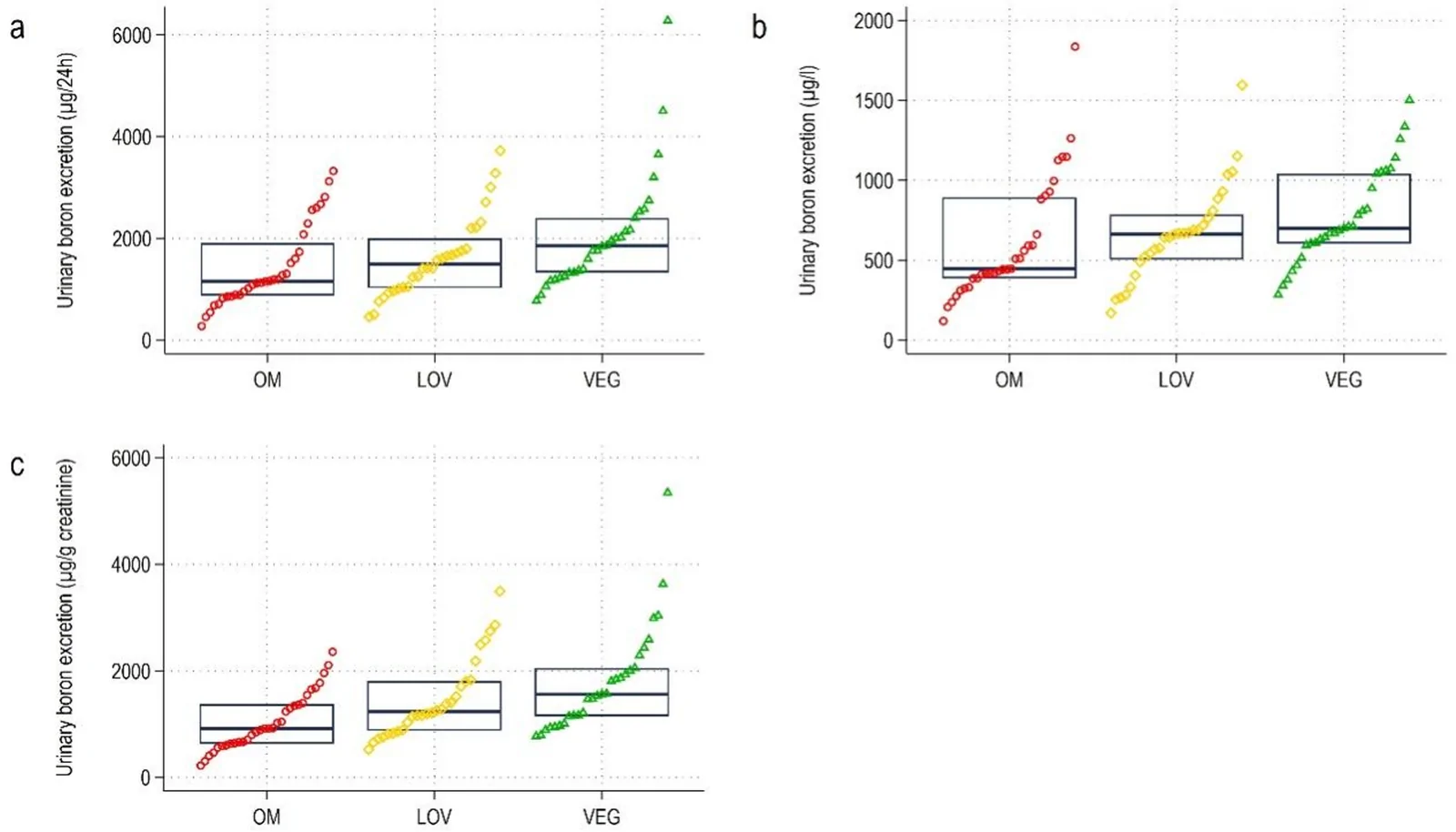
Twenty-four hour urinary boron excretion in *n* = 89 individuals participating in the German LAKE study by dietary group. OM = Omnivores. LOV = Lacto-ovo-vegetarians. VEG = Vegans. Panel a shows boron excretion in µg/24-h, whereas panel b shows concentrations (µg/l). Panel c displays a creatinine-corrected analysis. The between group difference was significant at *p* = 0.018 (panel a), *p* = 0.034 (panel b) and *p* < 0.001 (panel c). Boron excretion was highest in vegans, followed by lacto-ovo-vegetarians and omnivores in both analyses

Figure 2 displays urinary boron excretion in the study sample and by dietary group in a series of strip plots. Median 24-hour boron excretion in µg in the sample was 1415.40 (1053.61–2169.99). Median 24-hour boron excretion in µg/l in the sample was 642.96 (431.85–885.175). The median creatinine-corrected boron excretion in the entire sample was 1214.56 (863.96–1808.69) µg/g creatinine. Excretion was highest in vegans and lowest in omnivores (Fig. 2). Median 24-hour boron excretion concentration by diet group was as follows: 445.46 (386.82–893.36) µg/l in omnivores, 664.11 (506.88–786.34) µg/l in lacto-ovo-vegetarians and 700 (606.09–1042.23) µg/l in vegans. Creatinine-corrected median 24-hour boron excretion by diet group was as follows: 919.34 (639.18–1380.97) µg/g creatinine in omnivores, 1235.40 (877.956–1814.61) µg/g creatinine in lacto-ovo-vegetarians, and 1559.20 (1150.62–2057.23) µg/g creatinine in vegans. Between group differences were significant in the non-creatinine-corrected (*p* = 0.034) and the creatinine-corrected analysis (*p* < 0.001). We again observed a large interindividual variety in the sample. The lowest excretion was found in an omnivorous participant (222.73 µg/g creatinine) and the highest excretion was found in a vegan participant (5346.10 µg/g creatinine).

**Fig. 3:**
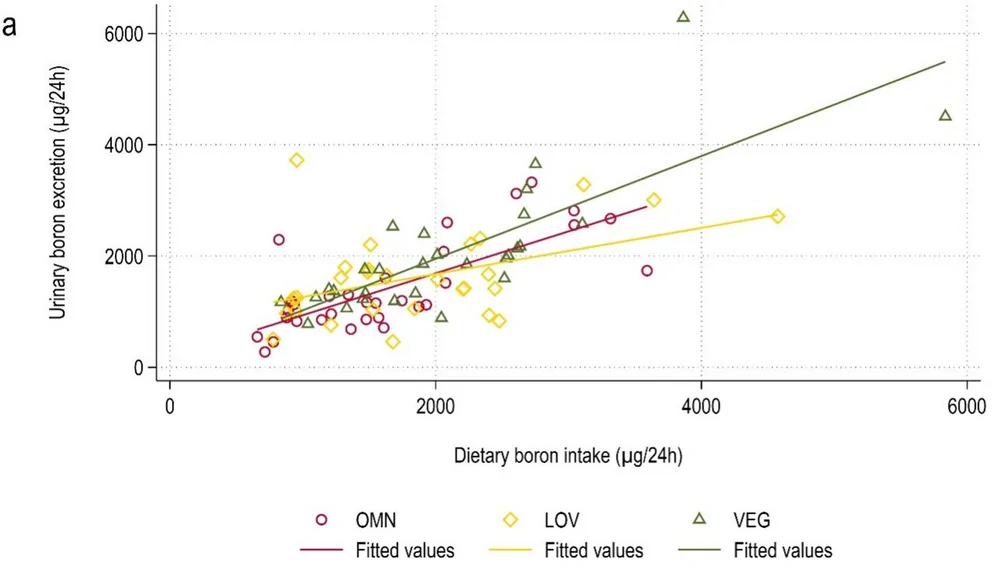
Correlation between dietary boron intake (in µg/24) and 24-hour urinary excretion (in µg) in *n* = 89 individuals participating in the German LAKE study. OMN = Omnivores. LOV = Lacto-ovo-vegetarians. VEG = Vegans. Correlation coefficients were as follows: *r* = 0.74 in omnivores, *r* = 0.45 in lacto-ovo-vegetarians and *r* = 0.79 in vegans, *p* < 0.001 for all, except for lacto-ovo-vegetarians (*p* = 0.016)

A strong positive correlation between dietary boron intake and urinary excretion was found in the whole sample and in all three diet groups (Fig. 3). Correlation coefficients were as follows: *r* = 0.74 in omnivores, 0.45 in lacto-ovo-vegetarians and 0.79 in vegans, *p* < 0.001 for all, except for lacto-ovo-vegetarians (*p* = 0.016).

**Fig. 4:**
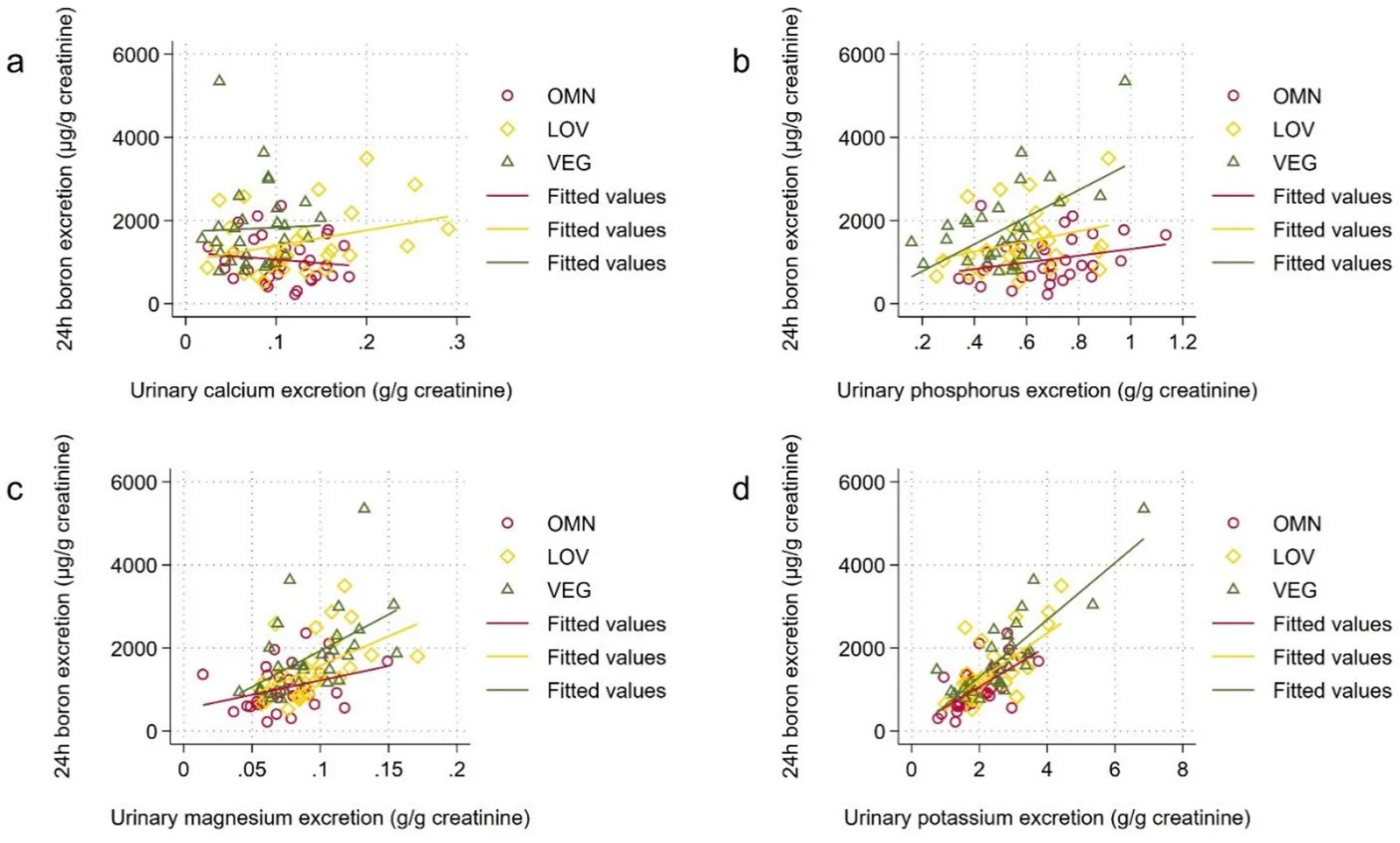
Correlation between 24-hour urinary boron excretion and the excretion of calcium (panel A), phosphorus (panel B), magnesium (panel C) and potassium (panel D) in *n* = 89 individuals participating in the German LAKE study. OMN = Omnivores. LOV = Lacto-ovo-vegetarians. VEG = Vegans. As for the calcium excretion in panel a, we found no significant correlations (*p* > 0.05 for all). A moderate association between the phosphorus and boron excretion was found (*r* = 0.57, *p* < 0.001). Significant associations between the magnesium and boron excretion were found in vegetarians (*r* = 0.49, *p* = 0.008) and in vegans (*r* = 0.52, *p* = 0.004). For potassium, significant associations were found in all dietary groups: omnivores: *r* = 0.64; vegetarians *r* = 0.65; vegans: *r* = 0.83)

Urinary boron excretion was also associated with the excretion of other chemical elements as displayed in Fig. 4. Positive associations were found with potassium (*r* ranging from 0.64 in omnivores to 0.83 in vegans, *p* < 0.001 for all). As for phosphorus, a significant association was only found in vegans (*r* = 0.57, *p* = 0.001). No significant associations were found with calcium excretion.

## Discussion

This study was conducted to test the hypothesis that boron intake and excretion differ between three popular dietary patterns, namely the vegan diet, the lacto-ovo-vegetarian diet and the omnivorous diet. Our results suggest that vegans had the highest dietary boron intake, although between-group differences were not statistically significant potentially due to the small sample size. Twenty-four-hour urinary boron excretion was highest in vegans, followed by lacto-ovo-vegetarians and omnivores. Dietary intakes and urinary excretion were significantly correlated in all diet patterns.

At this stage, there are no widely accepted RDAs or AIs for boron because a definite human requirement is yet to be established [10, 11]. Extrapolation of animal experiment data suggests that a daily intake of at least 1 mg of boron would achieve the nutritionally beneficial effects described in the literature [11]. In this sample, 82% of all participants achieved this goal. More specifically, 71% of omnivores, 78% of lacto-ovo-vegetarians and 97% of vegans reached this target intake. On the other hand, not a single participant exceeded the EFSA tolerable upper intake level for boron, which is currently set at 10 mg/day.

Boron is theoretically of high interest in lacto-ovo-vegetarians in vegans due to its potential effects on bone mass and bone health. Bone mass is a recurrent critical topic in lacto-ovo-vegetarian and vegan diets, with some studies suggesting an increased risk for osteoporotic fractures in plant-based diets [26]. Against this background, it appears of high interest that boron supplementation was suggested to be useful to support bone health (by preventing and maintaining adequate bone mineral density through controlling calcium, vitamin D and sex steroid hormone metabolism) in various populations [27]. A daily boron supplementation dose of 3 mg/d was suggested to exert beneficial effects in several studies. In the examined study population, none of the participants would have exceeded the EFSA tolerable upper intake level even when additionally adding the aforementioned 3 mg in the form of a supplement. Whether supplementing boron would result in additional bone protective effects, however, remains to be examined in future studies. The fact that boron supplementation was shown to markedly reduce urinary calcium excretion is another exciting phenomenon from a perspective on lacto-ovo-vegetarian and vegan diets and bone health [28]. Notably, the present study revealed no such associations. However, it appears noteworthy that boron excretion was associated with phosphorus excretion in vegans only. Excessive dietary phosphorus may by itself contribute to the burden of osteoporosis [29]. The fact that a higher boron excretion is associated with a higher phosphorus excretion in vegans is not directly supported by the currently available literature. A study by Nielsen et al. suggested that in the context of a low-magnesium diet in postmenopausal women, phosphorus excretion is effectively reduced by boron supplementation [30]. This was, however, not the case in women with an adequate magnesium intake [30]. Context-dependent, boron supplements may reduce urinary losses of phosphorus. The purported usage of boron supplements to impede the loss of phosphorus was also emphasized by Kjølholt et al., who highlighted that individuals often take boron supplements to prevent urinary calcium and magnesium loss [31]. As for phosphorus excretion in vegans, our results were not directionally consistent with this claim. Due to the cross-sectional nature of the study, however, no causal inferences can be made.

Notably, our results were directionally consistent with a 2015 study from Yunatskaya et al. [15]. Said study reported significantly higher median boron intakes in vegans and vegetarians as compared to omnivores in Omsk, Russia (1340.1 (758.5–2878.3) vs. 747.1 (747.1–1019.9 µg/d).

Future studies in lacto-ovo-vegetarians and vegans should nevertheless explore the role of boron in both diets. Whether supplementation could ensure an improved bone mass in lacto-ovo-vegetarian and vegan populations remains to be studies further. At this stage, our data suggest that a vegan diet may provide adequate dietary boron amounts. While almost all vegans met the 1 mg/d intake threshold, the number appeared less favorable in the remaining two diet patterns.

The present study has several strengths and weaknesses that warrant a brief discussion. As for the strengths, we report – *to the best of our knowledge* – the first study that specifically examined both boron intake and excretion in long-term lacto-ovo-vegetarians and vegans in Germany. The herein employed weighed food diaries are the gold standard to adequately capture dietary intakes when it comes to special diets that include a lot of special foods (e.g., tempeh) which are usually not listed in standard questionnaires. The study, however, was limited to two 24-hour dietary recording days. Additional days would have strengthened the study. As for the remaining weaknesses, we acknowledge that the sample size is only modest and that the sample is not representative of the German population. In fact, the sample was largely characterized by young participants with a high educational level, which may introduce a selection bias. The sample was likely not powered to detect small but significant between group intake differences. Secondary to that, recall bias and reporting bias regarding the weighed food diaries may not be excluded. Collecting 24-hour urine samples is challenging and could have influenced participants’ food intake during the second day of the study. The herein presented calculations are based on US data (e.g., the Rainey database [4]) but were supplemented with data from Choi and Yun [21] to adequately capture the higher amount of soy-based foods in the vegan diet. Comparable German data was not available, which is an important drawback of this study. Cross-sectional in nature, the study was ultimately not designed to make any causal inferences. A replication of our results in larger, prospective trials is thus warranted. Despite these limitations, however, we believe that our results add to the literature and help to better understand and quantify boron intakes in lacto-ovo-vegetarians and vegans in Germany.

## Conclusions

This study suggested that dietary boron intake and excretion differ between vegans, lacto-ovo-vegetarian and omnivores. In this sample, a vegan diet was suitable to reach the 1 mg intake target proposed earlier.

## Supplementary Information

Below is the link to the electronic supplementary material.

Supplementary Material 1 (DOCX 176 KB)

## Data Availability

The datasets used and analyzed during the current study are available from the corresponding author (MAS) on reasonable request.

## Abbreviations

AIs: Adequate Intakes
EFSA: European Food Safety Authority
LAKE: Load of Acid to Kidney Evaluation
LOV: Lacto-Ovo-Vegetarians
OM: Omnivores
RDAs: Recommended Dietary Allowances
VEG: Vegans
