# Supplementary material for: Boron Intake and 24-hour Urinary Excretion in Long-term Omnivores, Vegetarians and Vegans: a Cross-sectional Study
Source: Biol Trace Elem Res. 2026 Aug 12;204(11):8758–67. doi: 10.1007/s12011-026-05297-x (PMC13714143; doi:10.1007/s12011-026-05297-x)
Supplement: Supplementary file 1 [file 12011_2026_5297_MOESM1_ESM.docx]

Supplementary Figures

Supplementary Fig. 1 title: Participant inclusion flowchart

Supplementary Table 1 legend: OMN = Omnivores. LOV = Lacto-ovo-vegetarians. VN = Vegans.
